# Increasing operational and scientific efficiency in clinical trials

**DOI:** 10.1038/s41416-020-0990-8

**Published:** 2020-07-21

**Authors:** Deirdre Kelly, Anna Spreafico, Lillian L. Siu

**Affiliations:** Princess Margaret Cancer Centre, University Health Network, University of Toronto, Toronto, ON Canada

**Keywords:** Drug development, Clinical trial design

## Abstract

Operational and scientific inefficiencies in clinical trials represent roadblocks that need to be identified and circumvented to advance drug development in oncology. The collaboration of key stakeholders to advance this agenda is crucial to accelerate clinical research and ultimately benefit patient care through the optimal allocation of time and resources.

## Main

Clinical trials represent one of the most important mechanisms to translate scientific discoveries into clinical knowledge, evaluate new diagnostic or therapeutic regimens, and advance practice beyond the standard of care. The financial cost for conducting pivotal clinical trials of novel oncology therapeutic drugs that formed the basis for the United States Food and Drug Administration approval in 2015–2017 was estimated at 31.7 million (median cost per trial).^1^ The time and efforts expended on clinical trials by trial participants and study teams are much more difficult to quantitate. These tangible and intangible costs rise as clinical trials become increasingly more complex and arduous, underscoring an urgent need to examine the ways in which their efficiency can be optimised.

### From concept development to protocol activation

In 2006, Dilts et al.^2^ demonstrated that, based on 13 Phase 3 studies conducted under the auspices of a cooperative group, it took >370 distinct processes and a median duration of 784 days (range 537–1130 days) to activate a trial from the initial concept. In 2008, the United States National Cancer Institute (NCI) established an Operational Efficiency Working Group (OEWG) to identify the strategies to expedite trial activation, including setting target timelines for each step, promoting developmental processes to occur in parallel, and streamlining the contract and financial review procedures.^3^ The NCI invested in transformative measures such as centralised infrastructure and biomarker support to enhance drug development processes.^4^ For instance, a Central Institutional Review Board Initiative enabled a coordinated approach to human subject protection for national multicentre cancer treatment trials. Currently, the OEWG clock starts from the actual initial concept evaluation and stops at trial opening to enrolment, with absolute deadlines of 400–450 days for Phase 1 or 2 concepts and 540 days for Phase 3 concepts. The start-up timelines for industry-sponsored trials are less transparent to the public, but are closely scrutinised internally within pharmaceutical companies due to financial pressures such as those arising from the competitive environment and concerns regarding patent expiry. The urgency to speed up clinical trial processes has led to the launch of TransCelerate Biopharma in 2012, a non-profit organisation that aims to address critical challenges and establish harmonisation and sharing of the data, with prime examples being a shared investigator database and a common investigator curriculum vitae template that is accepted by all of its biopharmaceutical members.

### Enrolment stage

Multiple factors can lead to slow and inefficient enrolment of research subjects into clinical trials, such as unnecessarily strict eligibility criteria, burdensome trial requirement for visits and investigations beyond what would be appropriate for safety monitoring and endpoint evaluation, and the inflexibility of trial stipulations.^5^ Recruitment of under-represented patient populations into clinical trials represents an important strategy to accelerate study completion, but outreach efforts to fulfil this mandate must be prioritised and sustained. In the contemporary artificial intelligence era, tools that can match potentially eligible subjects to clinical trials are emerging as a reality.^6^ Some adaptations made during the coronavirus disease 2019 (COVID-19) pandemic may become permanent considerations to increase the clinical trial efficiency, while maintaining the principles of good clinical practice and pharmacovigilance. Processes and procedures amenable to modifications include the application of validated electronic signatures to replace wet-ink signatures, administration of parenteral trial treatments associated with low adverse event risks in local medical centres, and remote study monitoring.

### Enrolment completion, follow-up and closure stage

Perhaps the least attention has been paid to increasing the clinical trial efficiency at the stage when enrolment has been completed. Typically, after the recruitment activity has ceased, study participants are followed for a specific period of time for their vital status, and ultimately the final database lock and trial closure ensue. In a retrospective review of the early-phase clinical trials conducted by our Princess Margaret Cancer Centre Phase 1 Trials Programme from 2013 to 2019, 44 trials had patients in active survival follow-up. Of these, 30 (68%) trials mandated prolonged surveillance till death. Of the 552 patients enrolled in these 44 trials, 116 (21%) were in active survival follow-up with a median duration of 6.5 (range 1–48) months and had a median of 4 follow-up encounters (range 0–65). These data demonstrate that clinical trial protocols often remain open for prolonged periods of time despite all patients having completed the study treatment, thus substantially increasing the administrative burden at the trial sites. One approach to enable appropriate tracking is to transfer all patients to a single institution-based follow-up protocol, thus minimising the need to keep numerous study files active and repeatedly renewed. Some pharmaceutical partners have adopted such a practice to establish “roll-over” protocols to consolidate the follow-up requirements of multiple studies that shared the same sponsor. Lastly, the storage of clinical trial documents to fulfil regulatory requirements should be replaced by digitally scanned electronic formats. This conversion from paper into bytes would not only save time whenever access to archived files is needed in the future but also constitute an environmentally friendly solution.

### Scientific efficiency

In addition to the abovementioned ways to improve the operational efficiency of clinical trials, scientific strategies must be in place to maximise their chances of success while utilising the least patient resources and infrastructure. Only the most promising drug candidates should be selected for clinical testing, duplicative efforts without a strong justification (such as “me-too” trials) should be discouraged, and a high bar must be set for go-no-go decisions to launch large randomised registrational studies. Innovative clinical trial designs such as adaptive studies have pre-specified allowances to alter their enrolment criteria, and thus offer unique advantages by adjusting dynamically to changes in the existent therapeutic landscape. Biomarkers, especially those of sensitivity or resistance, are critical in accelerating clinical research by selecting patients who are most or least likely to benefit; yet, the current pace of biomarker discovery and validation is slow. To a large extent, the scientific inefficiency in clinical trials is attributable to the lack of data sharing of pre-competitive and post-competitive information, such as early nonclinical evaluations and individual patient-level clinical and biomarker results in published clinical trials, respectively.

### Conclusion

The optimisation of operational and scientific efficiency in clinical trials requires concerted efforts from investigators and study teams, sponsors, regulatory agencies, and other key stakeholders. The elimination of bottlenecks is critical to conserve resources and time, and, importantly, to accelerate progress in cancer research.

## Data Availability

Not applicable.
